# Graphene Oxide‐Supported Chromium‐Containing Polyoxometalate as an Efficient Heterogeneous Catalyst for Nitroarene Reduction Under Mild Conditions

**DOI:** 10.1002/gch2.70159

**Published:** 2026-09-22

**Authors:** Maryam Khalaji‐Verjani, Majid Masteri‐Farahani, Alireza Badiei, Ghodsi Mohammadi Ziarani, Senem Akkoc, Mehran Feizi‐Dehnayebi

**Affiliations:** ^1^ School of Chemistry, College of Science University of Tehran Tehran Iran; ^2^ Faculty of Chemistry Kharazmi University Tehran Iran; ^3^ Department of Organic Chemistry, Faculty of Chemistry Alzahra University Tehran Iran; ^4^ Department of Basic Pharmaceutical Sciences, Faculty of Pharmacy Suleyman Demirel University Isparta Turkey; ^5^ Faculty of Engineering and Natural Sciences Bahcesehir University Istanbul Turkey; ^6^ Department of Chemical Engineering, Engineering Faculty Suleyman Demirel University Isparta Turkey

**Keywords:** chromium‐containing polyoxometalate, graphene oxide, heterogeneous catalysis, nitroarene, reduction

## Abstract

Selective hydrogenation of nitroarenes is a valuable organic reaction that has attracted significant interest from researchers in recent years. Herein, a new nanocomposite (GO@POM‐Cr) was prepared via an electrostatic assembly method. The surface of graphene oxide (GO) was modified with ammonium groups, and the chromium‐containing polyoxomolybdate anion, Na_4_[PCr(H_2_O)Mo_11_O_39_] (POM‐Cr), was subsequently immobilized on GO as the counter anion to the ammonium cation through a simple ion‐exchange process. The Fourier‐ transform infrared (FT‐IR) spectroscopy confirmed the successful immobilization of POM‐Cr, with the P‐O_a_ peak at ∼1118 cm^−1^ appearing as a single, unsplit band, directly evidencing chromium incorporation into the Keggin framework. The surface properties of GO@POM‐Cr were further investigated using Raman spectroscopy, X‐ray diffraction (XRD) analysis, and elemental analysis. Remarkably, the GO@POM‐Cr nanocomposite demonstrated high catalytic efficiency for nitroarene reduction (99% conversion) under mild reaction conditions. The reactions were performed in ethanol as a green solvent in the presence of hydrazine hydrate as the reducing agent. Mechanistic investigations suggested that the reduction proceeds via a direct pathway involving nitroso and hydroxylamine intermediates, affording the corresponding anilines with excellent selectivity and catalyst stability over multiple reaction cycles.

## Graphical abstract

A chromium‐substituted monovacant Keggin polyanion was immobilized on the surface of graphene oxide. The obtained nanocatalyst showed high catalytic activity in the reduction of various nitroarenes under mild conditions. GC‐MS analysis confirmed the direct pathway for reduction of nitroarenes in the presence of the catalyst.

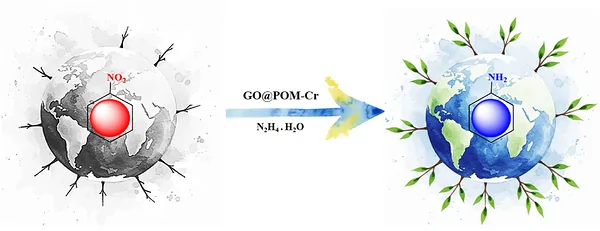

## 1 Introduction

Catalytic reduction of nitrobenzene and its derivatives is one of the most important reactions in academic research, as these compounds are a source of environmental pollution [1]. Moreover, the products of this reaction are highly valuable and widely used in industrial production. The nitroarene compounds are generated as by‐products during combustion of fossil fuel in various industrial processes. The catalytic reduction of these toxic compounds into value‐added products significantly contributes to reducing environmental pollution and supporting economic growth. The obtained anilines produced from this reaction are valuable compounds that are widely used in various industries, including pharmaceuticals, dyes, fragrances, and agrochemicals [2, 3]. These applications have made the reduction of nitroarenes into aromatic amines an important target in the fields of industrial chemistry and environmental protection. In this regard, non‐noble metal‐based catalysts, such as Cu, Co, and Ni have attracted special attention as suitable alternatives due to their lower cost and greater availability. So far, the nitroarenes reduction has been carried out using various catalytic systems.

In recent years, polyoxometalates (POMs) have attracted significant attention as green catalysts for organic transformations [4]. The POMs are anionic metal oxide clusters composed of transition metal atoms and oxygen [5, 6]. Transition metals typically have a d^0^ electronic configuration, and the oxygen atoms occupy bridging and terminal positions within the structure. POMs constitute a large and structurally diverse family of compounds, exhibiting a wide range of compositions and properties. As an important branch of POMs, transition metal‐substituted POMs have a well‐defined structure. In such POMs, the incorporated transition metals are typically coordinated in an octahedral environment in which one of the coordination sites is occupied by a solvent molecule. In most cases, the aquametal (III) species can be reduced to aquametal (II) and oxidized to the corresponding oxometal (IV), hydroxometal (IV), and oxometal (V) derivatives depending on the specific properties of the incorporated metals [7]. These compounds, due to their unique characteristics, have been applied in different fields such as medicine, electronics, adsorption, and catalysis. These properties include high sustainability, shapes and sizes, redox potentials, variety of compositions, and non‐toxicity [8, 9, 10, 11, 12, 13].

Upon combining with solid supports such as graphene oxide (GO) or graphitic carbon nitride (C_3_N_4_), POMs can create efficient hybrid catalytic systems with improved stability, accessibility of active sites, and catalytic performance. These solid supports facilitate the development of POM‐based composites by providing suitable anchoring sites, preventing aggregation of POM species, and improving the overall properties of the catalytic system, including electron transfer ability and structural stability [14]. However, the immobilization of transition metal‐substituted Keggin‐type POMs on carbon‐based supports for nitroarene reduction remains relatively less explored. In this study, we report the synthesis of a chromium‐containing Keggin‐type polyoxometalate through a simple incorporation strategy and its immobilization onto the surface of GO using aminopropyltrimethoxysilane (APTS) as an organic linker. The prepared GO@POM‐Cr composite was designed as a heterogeneous catalyst to combine the redox properties of the Cr‐containing POM with the structural advantages of the GO support. The catalytic activity of the synthesized composite was investigated in the reduction of a series of nitroarenes to their corresponding anilines under mild reaction conditions. This work provides a new POM‐based hybrid catalytic system with improved recyclability and efficient catalytic performance for nitroarene reduction.

## 2 Experimental Section

The details of the used materials and instruments can be found in the Supporting Information.

### 2.1 Synthesis of Na_4_[PCr(H_2_O)Mo_11_O_39_], (POM‐Cr)

**FIGURE 1:**
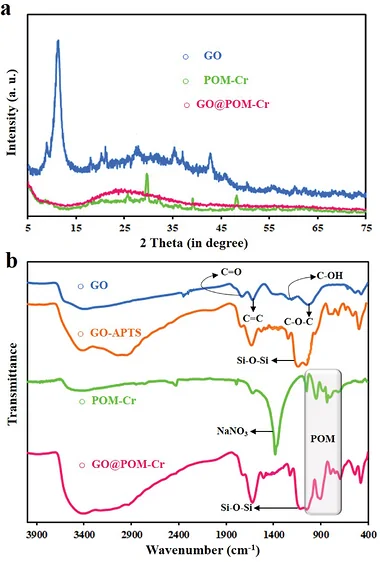
(a) XRD patterns of GO, POM‐Cr, and GO@POM‐Cr and (b) FT‐IR spectra of GO, GO‐APTS, POM‐Cr, and GO@POM‐Cr.

The synthesis method for Molybdochromophosphate (POM‐Cr) is based on modified procedures reported in the literature [15, 16, 17]. In the first step, 5 mmol of Na_2_MoO_4_·2H_2_O (1.3 g) and 0.5 mmol of Na_2_HPO_4_ (0.07 g) were dissolved in 30 mL of deionized (DI) water. In the second step, the temperature of the mixture was adjusted to 85°C and the mixture was stirred for 30 min. Subsequently, 3 mL of 3 M nitric acid was added dropwise in order to adjust the pH of the hot solution to approximately 4.3. Then, 0.5 mmol of Cr(NO_3_)_3_·9H_2_O (0.2 g) was added to the solution at a pH of 4.3. The reaction mixture was continuously stirred at 85°C for 1 h to ensure complete synthesis of Na_4_[PCr(H_2_O)Mo_11_O_39_]. Finally, the solid product Na_4_[PCr(H_2_O)Mo_11_O_39_] was obtained after cooling to room temperature, accompanied by the formation of NaNO_3_ as a by‐product (Figure 1b).

### 2.2 Synthesis of GO@POM‐Cr Catalyst

1 g of graphene oxide (GO) (prepared according to the literature method [18] as described in Section S1) was dispersed in 30 mL of toluene. Next, 1 mL of aminopropyltriethoxysilane (APTS) was added to the GO mixture, and the reaction mixture was refluxed under N_2_ atmosphere for 12 h. The solid product was washed with methanol to remove the excess of APTS. The resulting product was dispersed in 20 mL of 0.1 m HCl, which protonated the amine groups, forming GO‐Si (OR)_3_(CH_2_)_3_‐NH_3_^+^ (GO‐NH_3_^+^). This resulting solid was washed with deionized (DI) water and dried. Subsequently, 1 g of GO‐NH_3_^+^ was dispersed in water with an ultrasonic probe. 1 mmol of prepared POM‐Cr was dissolved in 20 mL of DI water and then added to the GO‐NH_3_^+^ dispersion. The mixture was continuously stirred at room temperature for 3 h to ensure complete interaction between the POM‐Cr species and the GO‐NH_3_^+^ surface. Afterward, the GO@POM‐Cr product was obtained by washing with cold DI water to remove NaNO_3_ as a by‐product and excess of POM‐Cr.

### 2.3 Catalytic Reduction of Nitroarenes in the Presence of GO@POM‐Cr Nanocomposite

The catalytic activity of the GO@POM‐Cr nanocomposite was investigated in the catalytic reduction of nitroarenes in ethanol (2 mL) as the solvent in a 5 mL glass vial. GO@POM‐Cr (20 mg), nitrobenzene (NB; 0.5 mmol, 50 µL), and the reductant (N_2_H_4_.H_2_O; 5 mmol, 307 µL) were added to ethanol to form the reaction mixture. The reduction reaction was carried out with stirring at 50°C. After 2 h, thin‐layer chromatography (TLC) analysis confirmed that all nitrobenzene had been converted to aniline. To investigate the stability and recyclability of the GO@POM‐Cr nanocomposite, after each test, the GO@POM‐Cr nanocomposite was separated, washed several times with ethanol, and dried in an oven before reuse in the next reaction cycles.

## 3 Results and Discussion

### 3.1 Preparation of GO@POM‐Cr

**SCHEME 1:**
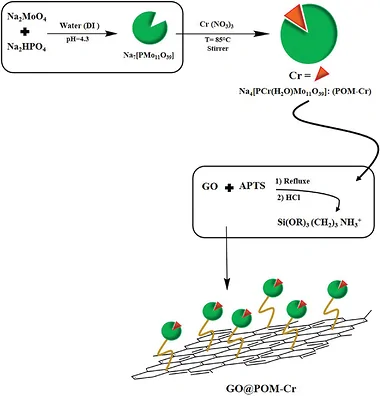
Schematic illustration of the formation process of the GO@POM‐Cr nanocomposite.

Scheme 1 illustrates the typical synthesis strategy for GO@POM‐Cr. After preparing the POM‐Cr clusters, they were immobilized on the surface of graphene oxide (GO) using APTS as a linker. The amino‐functionalized GO helps the anionic POM‐Cr species to attach efficiently, resulting in a uniform distribution of the clusters on the GO surface.

### 3.2 Characterization of GO@POM‐Cr

#### 3.2.1 XRD Analysis

Figure 1a illustrates the XRD patterns of GO sheets, the POM‐Cr, and the GO@POM‐Cr nanocomposite to determine the crystal phases. The GO sample shows a characteristic peak at 2θ = 11.27° assigned to the (001) plane, which shifts to a lower angle (2θ ≈ 5°) and becomes weaker in the GO@POM‐Cr nanocomposite. This shift indicates that the incorporation of the POM‐Cr polyanion increases the interlayer spacing of GO and disrupts its ordered lamellar structure [19]. Furthermore, the XRD pattern of the GO@POM‐Cr nanocomposite displays a broad diffraction peak at around 25°, which is a characteristic peak of POM‐Cr, suggesting the successful dispersion of POM‐Cr polyanions. This observation confirms that the POM‐Cr polyanions are well dispersed onto the GO sheets, consistent with the results of the elemental analysis (see Figure 4) [20].

#### 3.2.2 FT‐IR Spectroscopy

The FT‐IR spectra of GO exhibit absorption peaks at 1735, 1625, 1220, and 1050 cm^−1^, corresponding to the stretching vibrations of C═O, C═C, C─OH, and C─O─C groups, respectively, as shown in Figure 1b [21, 22]. Additionally, the FT‐IR spectrum of GO‐APTS shows a strong absorption peak around 1100 cm^−1^ assigned to Si─O─Si stretching vibration, which indicates the successful aminopropylsilylation of the GO surface [22]. The POM‐Cr polyanions exhibit characteristic peaks associated with the polyoxometalate Keggin family. The characteristic peaks appear at 1118, 950, 881, and 790 cm^−^^1^, corresponding to the P─O_a_ stretching vibration of the central tetrahedral oxygen, the terminal M═O_t_ metal–oxygen bond, the corner‐shared M‐O_b_‐M bridging vibration, and the edge‐shared M‐O_c_‐M vibration, respectively. These assignments highlight the distinct connectivity between the central and peripheral oxygen atoms and the metal centers within the Keggin framework [15, 23]. The band observed at 1380 cm^−^^1^ is attributed to the NaNO3 by‐product, which can be removed by subsequent washing steps [24, 25]. Notably, the P‐O_a_ peak at 1118 cm^−^^1^ appears as a single, unsplit band. In contrast, lacunary Keggin‐type structures typically exhibit a split of the P─O_a_ bonds into two peaks due to reduced symmetry around the phosphorus heteroatom. In this case, the coordination of chromium partially restores the local symmetry by bonding with the available oxygen atoms of phosphorus, leading to the observed single P─O_a_ band and confirming the integrity of the tetrahedral unit within the synthesized compound. This splitting is reduced by the presence of chromium in the structure, making it more similar to the complete polyoxometalate with higher symmetry. The unsplit nature of the P‐O_a_ peak thus serves as direct evidence for the successful incorporation of chromium into the Keggin framework. The FT‐IR spectrum of GO@POM‐Cr displays characteristic Si─O─Si stretching bands at 1100 cm^−1^ attributed to APTS, along with distinct vibrational modes associated with the POM‐Cr framework. Additionally, the spectrum reveals indicative peaks corresponding to GO, which partially overlap with those of APTS and POM‐Cr, making precise peak assignment more challenging [15, 26].

#### 3.2.3 Morphology Observation and Elemental Analysis

**FIGURE 2:**
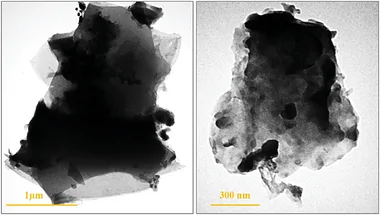
TEM images of GO@POM‐Cr.

**FIGURE 3:**
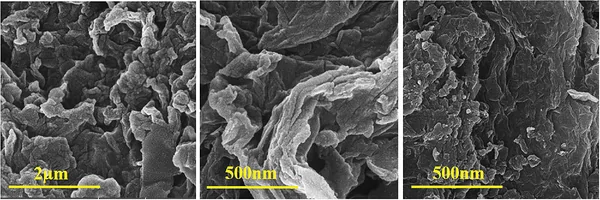
FE‐SEM images of GO@POM‐Cr.

To obtain detailed information regarding the morphology and elemental composition of the GO@POM‐Cr nanocomposite, transmission electron microscopy (TEM), field emission scanning electron microscopy (FE‐SEM), elemental mapping, and energy dispersive x‐ray (EDX) analyses were performed. TEM images of the GO@POM‐Cr composite (Figure 2) reveal a crumpled, wrinkled, and sheet‐like morphology, characteristic of functionalized GO nanosheets. The observed folded structure indicates that the GO framework was preserved after the incorporation of POM‐Cr species. As shown in Figure 3, the FE‐SEM images of the GO@POM‐Cr nanocomposite exhibit a wrinkled structure. This morphology is consistent with the presence of GO nanosheets in the synthesized nanocomposite [20, 27].

**FIGURE 4:**
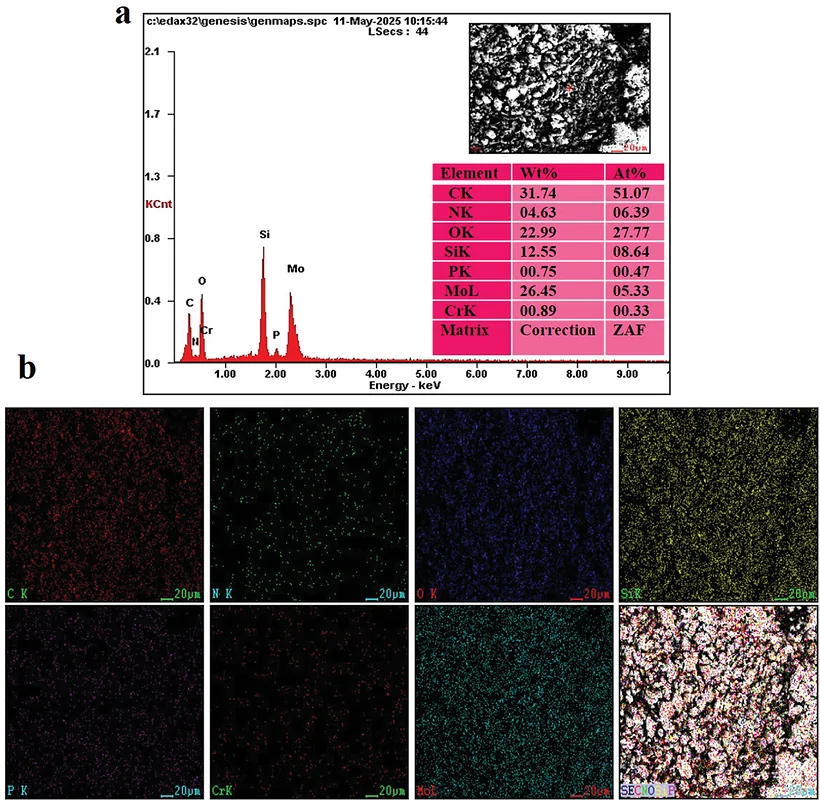
(a) EDX spectrum and (b) elemental mapping images of GO@POM‐Cr.

In addition, Figure 4a shows the elemental analysis of the GO@POM‐Cr nanocomposite, confirming the successful synthesis of the nanocomposite (as indicated by the peaks of Cr, P, Mo, O, N, Si, and C in the EDX spectrum). The elemental mapping images of the GO@POM‐Cr nanocomposite further support the EDX data (Figure 4a), demonstrating the uniform elemental distribution within the nanocomposite (Figure 4b).

#### 3.2.4 N_2_ Adsorption‐Desorption Analysis

**FIGURE 5:**
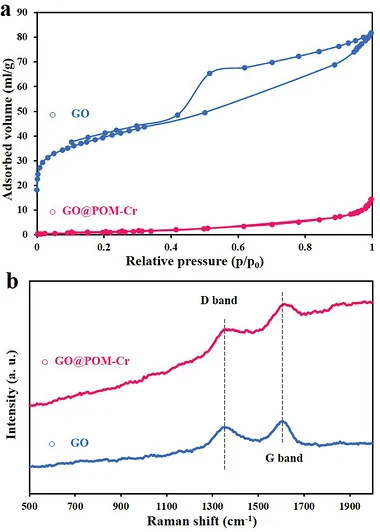
(a) N_2_ adsorption‐desorption isotherms and (b) Raman spectra of GO and GO@POM‐Cr.

N_2_ adsorption‐desorption isotherms of GO and GO@POM‐Cr nanocomposite are shown in Figure 5a. The GO isotherm corresponds to type‐IV, indicating the mesoporosity in GO as confirmed by the observation of a hysteresis loop, according to the BDDT (Brunauer–Deming–Deming–Teller) classification [28, 29]. After immobilization of POM‐Cr on GO, the GO@POM‐Cr nanocomposite exhibits a type‐II isotherm [30]. This result is related to a decrease in the mesoporosity of GO, which occurs due to the successful immobilization of POM‐Cr clusters on the GO surface, partially blocking the pores and reducing the overall accessible surface area. This process also affects the GO@POM‐Cr isotherm in another way: the surface area decreases from 302 to 4.49 m^2^ g^−1^ [20].

#### 3.2.5 Raman Spectroscopy

Figure 5b presents the Raman spectra of GO and the GO@POM‐Cr nanocomposite, showing a defect (D) band at 1342 cm^−1^ and a graphitic (G) band at 1580 cm^−1^. These bands correspond to the disordered graphitic carbon and the graphitization degree, respectively [31]. After the immobilization of the POM‐Cr polyanion onto the GO surface, the G and D bands remained, indicating the preservation of the GO structure in the synthesized GO@POM‐Cr nanocomposite. This preservation is attributed to the successful incorporation of POM‐Cr polyanion on the GO surface without affecting its main structure [32, 33].

### 3.3 Catalytic Nitroarene Reaction

**SCHEME 2:**
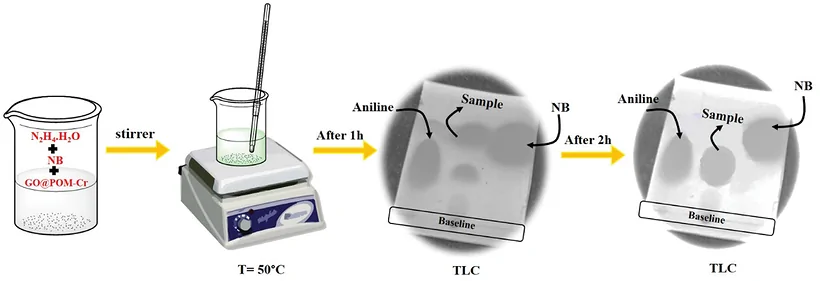
Reduction steps of the selected model under optimal conditions, monitored using the TLC technique.

To determine the optimal reaction conditions, we used nitrobenzene as a model substrate (Scheme 2). Then, by varying key parameters, the optimal conditions for the reduction of a range of nitroarenes were determined. The determination of the optimal amount of GO@POM‐Cr catalyst is presented in entries 1–4 of Table 1. Based on these results, 20 mg was found to be the optimal amount (time = 2 h). Ethanol was selected as the solvent because it provided better results for the reduction of nitrobenzene (entries 1, 5–7, Table 1). Temperature is an important parameter for this type of reaction, and the optimal temperature was found to be 50°C. To evaluate the effect of temperature, a reduction test was performed at room temperature (RT). After more than 10 h, the reaction was incomplete and unreacted nitrobenzene remained in the reaction vessel (entry 8, Table 1). To investigate the effect of N_2_H_4_.H_2_O as a reductant, various amounts were tested, as shown in entries 1,9–11 of Table 1. By decreasing the amount of N_2_H_4_.H_2_O, the time required to achieve 99% conversion increased. Similarly, when N_2_H_4_.H_2_O was not used, no conversion of nitrobenzene to aniline was observed. Therefore, the presence of N_2_H_4_.H_2_O was necessary for carrying out the reaction. To investigate the synergistic effect between the components of the synthesized catalyst GO@POM‐Cr, the raw POM‐Cr and GO were used as catalysts under optimal conditions (entries 12 and 13 of Table 1, respectively). These individual compounds were unable to reduce the nitrobenzene, indicating that GO and POM‐Cr exhibit a synergistic effect when combined. Entry 14 in Table 1 also demonstrates that the presence of GO@POM‐Cr is essential for the reduction reaction.

**TABLE 1:** Optimization of the reaction conditions for the reduction of nitrobenzene using GO@POM‐Cr.^a^

Entry	Catalyst	Solvent	N_2_H_4_.H_2_O [mmol]	Time [h]^b^
1	20 mg‐ GO@POM‐Cr	ethanol	5	2
2	10 mg‐ GO@POM‐Cr	ethanol	5	∼ 3
3	5 mg‐ GO@POM‐Cr	ethanol	5	∼ 4
4	1 mg‐ GO@POM‐Cr	ethanol	5	∼ 10
5	20 mg‐ GO@POM‐Cr	methanol	5	∼ 3.5
6	20 mg‐ GO@POM‐Cr	propanol	5	∼ 4
7	20 mg‐ GO@POM‐Cr	acetonitrile	5	∼ 2.5
8	20 mg‐ GO@POM‐Cr ^c^	ethanol	5	>10
9	20 mg‐ GO@POM‐Cr	ethanol	1	∼ 6.5
10	20 mg‐ GO@POM‐Cr	ethanol	2	∼10
11	20 mg‐ GO@POM‐Cr^d^	ethanol	0	−
12	20 mg‐ POM‐Cr^d^	ethanol	5	−
13	20 mg‐GO^d^	ethanol	5	−
14	None^d^	ethanol	5	−

^a^ Reaction conditions: Nitrobenzene (0.5 mmol), ethanol (2 mL), N_2_H_4_.H_2_O (5 mmol), temperature (50°C), conversion (99%).

^b^ Time necessary for complete conversion (99%).

^c^ The reaction remained incomplete after 10 h in room temperature.

^d^ The reaction showed no conversion.

### 3.4 Catalytic Reduction of Functionalized Nitroarenes

One of the important parameters in evaluating the value of a catalyst is its catalytic properties and scope of application. Therefore, we investigated the performance of this synthetic heterogeneous catalyst by reducing various nitroarenes. Based on the obtained optimal conditions, several nitroarenes with different electron‐withdrawing and electron‐donating substitutions were examined (Table 2). As previously mentioned, nitrobenzene was employed as a model substrate and exhibited complete conversion (99%) to aniline within 2 h. The study of other nitroarene substrates revealed that the nature of the substituents significantly influences the reduction outcomes. It is evident that electron‐withdrawing groups enhance the rate of the reduction reaction compared to electron‐donating groups. As shown in Table 2 (entries 2–6), halogen and other electron‐withdrawing substituents achieved 99% conversion in shorter reaction times. In contrast, substrates bearing electron‐donating groups required longer times to reach completion (Table 2, entries 7 and 8).

**TABLE 2:** Reduction of various nitroarenes in the presence of GO@POM‐Cr.^a^

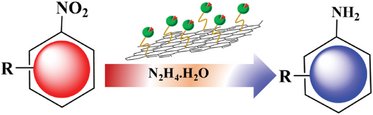			

Entry	Substrates	Product	Time [h]^b^
1	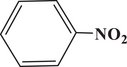	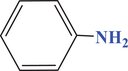	2
2	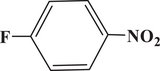	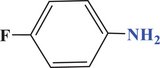	2
3	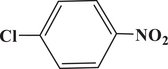	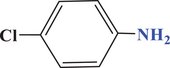	2.5
4	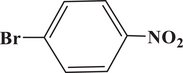	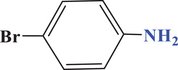	2.5
5	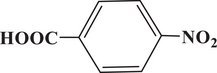	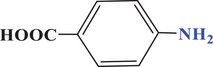	2
6	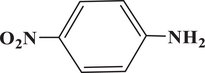	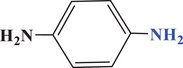	2
7	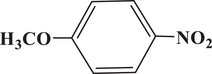	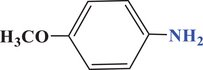	4
8	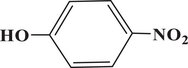	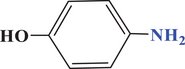	5

^a^ Reaction conditions: nitroarene (0.5 mmol), ethanol (2 mL), N_2_H_4_.H_2_O (5 mmol), catalyst (20 mg; with respect to initial substrate, temperature (50°C), and conversion (99%).

^b^ Time necessary for complete conversion (99%).

### 3.5 Reusability

**SCHEME 3:**
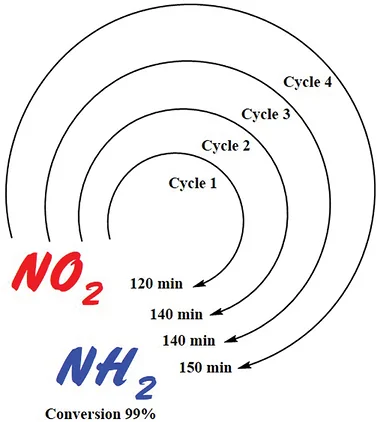
Catalytic reusability of GO@POM‐Cr in the reduction of nitrobenzene over consecutive four cycles.

The reusability of the GO@POM‐Cr catalyst was examined through four successive reduction cycles of nitrobenzene (Scheme 3). After each use, the catalyst was washed with ethanol, separated by centrifugation, and subsequently dried in an oven. Although the obtained results indicated a decrease in conversion over time, this reduction was insignificant, as 99% conversion was still achieved within 150 min.

### 3.6 Comparison With Previously Reported Catalysts

To evaluate the performance of the synthesized GO@POM‐Cr nanocomposite, a comparison with previous studies is presented in Table 3. The cases listed in Table 3 were conducted under various nitroarene reduction conditions, such as visible light irradiation (entries 1 and 2), the presence of hydrogen gas as a hydrogen source (entry 3), or different temperatures (entries 4–6). Based on this comparison, it can be concluded that the synthesized catalyst exhibits relatively superior performance in the reduction of nitroarenes under mild conditions (entry 6).

**TABLE 3:** Comparison of catalytic performances in nitrobenzene reduction to aniline over different catalysts.

Entry	Catalyst	Temp [°C]	Reductant	Solvent	Time [h]	Conv. [%]	Refs.
1	[Na_0.17_Rh_0.83_^III^(C_6_H_8_N_2_)_2_Cl_2_]_2_(C_8_H_8_N_2_)_2_ [As_4_W_40_O_140_Rh_4_^IV^(C_6_H_4_N_2_S)_2_] ·*n*H_2_O	Visible light	N_2_H_4_·H_2_O	Methanol	6	96.2	[34]
2	NaH_2_[Ru(bpy)_2_Cl_2_O (PW_11_O_39_)].10H_2_O	Visible light	N_2_H_4_·H_2_O	H_2_O/Ethanol	12	99.8	[35]
3	1.41%Pd/Ni‐SiW	40	H_2_	Ethanol	4.5	99	[36]
4	[Sb_8_Mo^VI^_13_MoV_5_O_66_]^5–^	80	N_2_H_4_·H_2_O	Ethanol	2	100	[37]
5	[Mo^IV^_6_ Mo^VI^_7_ O_32_(OH)_4_py_6_] ^2−^	80	N_2_H_4_·H_2_O	Ethanol	2	100	[38]
6	GO@POM‐Cr	50	N_2_H_4_·H_2_O	Ethanol	2	99	This work

### 3.7 Mechanism

**SCHEME 4:**
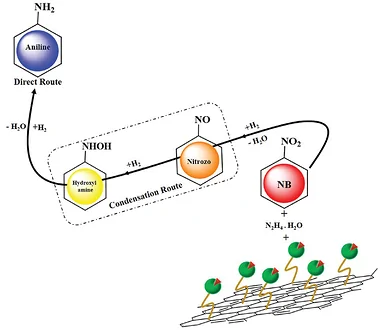
Proposed direct reaction pathway for the reduction of the nitro group to the amino group with GO@POM‐Cr catalyst.

In 1898, Haber proposed the first mechanism for the reduction of nitroarenes, which includes two pathways: direct and condensation [39, 40]. We used gas chromatography‐mass spectrometry (GC‐MS) analysis to investigate the mechanism (Figure S1). The condensation and direct pathways proceed through the hydrogenation of azobenzene and hydroxylamine intermediates, respectively [41]. In our proposed reduction reaction, hydrogen is supplied by the dissociation of hydrogen atoms from N_2_H_4_.H_2_O [42]. The azobenzene intermediate in the condensation pathway is stable and was not observed in GC‐MS analysis. Therefore, it can be concluded that the reduction reaction proceeds via the direct pathway [43] (Scheme 4).

## 4 Conclusion

In this study, a novel GO@POM‐Cr nanocomposite was fabricated for the reduction of nitroarenes. The GO@POM‐Cr catalyst was synthesized by immobilizing POM‐Cr on the GO surface through an APTS organic linker. Characterization techniques confirmed the functionalization steps and successful immobilization of POM‐Cr species onto the support surface. The catalyst system exhibited the properties of a heterogeneous catalyst with the possibility of easy separation. The GO@POM‐Cr demonstrated remarkable catalytic activity, achieving 99% conversion for the reduction of various nitroarenes to their corresponding aminoarenes using N_2_H_4_.H_2_O.

## Author Contributions

**Maryam Khalaji‐Verjani**: Investigation, Methodology, Writing – original draft. **Majid Masteri‐Farahani**: Software, Investigation, Formal analysis. **Alireza Badiei**: Conceptualization, Formal analysis, Writing – review & editing. **Ghodsi Mohammadi Ziarani**: Investigation, Methodology, Formal analysis, Writing – review & editing. **Senem Akkoc**: Validation, Software, Writing – review & editing. **Mehran Feizi‐Dehnayebi**: Writing – review & editing.

## Conflicts of Interest

The authors declare no conflicts of interest.

## Supporting information




**Supporting File**: gch270159‐sup‐0001‐SuppMat.docx.

## Data Availability

The data that support the findings of this study are available on request from the corresponding author

## References

[1] Y. Jiang , C. Chen , K. Li , L. P. Cui , and J. Chen , “Polyoxometalates for the Catalytic Reduction of Nitrogen Oxide and Derivatives: From Novel Structures to Functional Applications,” Chemical Communications 61 (2025): 4881–4896. 10.1039/d5cc00632e 40062997

[2] F. Chen , H. Yan , J. Wang , et al., “High‐Efficient Catalytic Ozonation for Degradation of Nitrobenzene in Water With Ce‐Doped LaCoO_3_ Catalyst,” Journal of Materials Science 59 (2024): 3406–3420, 10.1007/s10853-024-09437-3.

[3] G. Liu , C. Chen , and J. Chen , “Catalytic and Electrocatalytic Hydrogenation of Nitroarenes,” The Journal of Physical Chemistry C 127 (2023): 4375–4386, 10.1021/acs.jpcc.3c00270.

[4] M. Malmir , M. M. Heravi , Z. Yekke‐Ghasemi , and M. Mirzaei , “Incorporating Heterogeneous Lacunary Keggin Anions as Efficient Catalysts for Solvent‐free Cyanosilylation of Aldehydes and Ketones,” Scientific Reports 12 (2022): 11573, 10.1038/s41598-022-15831-1. PMC9262904 35799059

[5] A. Misra , K. Kozma , C. Streb , and M. Nyman , “Beyond Charge Balance: Counter‐Cations in Polyoxometalate Chemistry,” Angewandte Chemie International Edition 59 (2020): 596–612, 10.1002/anie.201905600. PMC6972580 31260159

[6] E. Hampson , J. M. Cameron , S. Amin , et al., “Asymmetric Hybrid Polyoxometalates: A Platform for Multifunctional Redox‐Active Nanomaterials,” Angewandte Chemie 131 (2019): 18449–18453, 10.1002/ange.201912046. PMC6916258 31595597

[7] M. Sadakane and E. Steckhan , “Electrochemical Properties of Polyoxometalates as Electrocatalysts,” Chemical Reviews 98 (1998): 219–238, 10.1021/cr960403a. 11851504

[8] J. H. Li , X. L. Wang , G. Song , H. Y. Lin , X. Wang , and G. C. Liu , “Various Anderson‐Type Polyoxometalate‐Based Metal–Organic Complexes Induced by Diverse Solvents: Assembly, Structures and Selective Adsorption for Organic Dyes,” Dalton Transactions 49 (2020): 1265–1275, 10.1039/C9DT04397G. 31904036

[9] J. Wang , Z. Tao , T. Tian , et al., “Polyoxometalate Nanoclusters: A Potential Preventative and Therapeutic Drug for Inflammatory Bowel Disease,” Chemical Engineering Journal 416 (2021): 129137, 10.1016/j.cej.2021.129137.

[10] Z. Liu , Q. Chang , W. Wu , et al., “Bridging Polyoxometalate‐Based Mn_4_ Cubane Clusters With Inorganic Phosphates: Structural Transformation and Magnetic Properties,” Inorganic Chemistry 60 (2020): 219–224, 10.1021/acs.inorgchem.0c02837. 33320667

[11] J. Zhong , J. Pérez‐Ramírez , and N. Yan , “Biomass Valorisation Over Polyoxometalate‐Based Catalysts,” Green Chemistry 23 (2021): 18–36, 10.1039/D0GC03190A.

[12] N. Aramesh , B. Yadollahi , and V. Mirkhani , “Fe(III) Substituted Wells–Dawson Type Polyoxometalate: An Efficient Catalyst for Ring Opening of Epoxides With Aromatic Amines,” Inorganic Chemistry Communications 28 (2013): 37–40, 10.1016/j.inoche.2012.11.005.

[13] X. Wu , T. Huang , Q. Wu , and L. Xu , “Synthesis and Conductive Performance of Indium‐Substituted Ternary Heteropoly Acids With Keggin Structures,” Dalton Transactions 45 (2016): 271–275, 10.1039/C5DT02541A. 26597714

[14] D. K. Rajak , D. D. Pagar , R. Kumar , and C. I. Pruncu , “Recent Progress of Reinforcement Materials: A Comprehensive Overview of Composite Materials,” Journal of Materials Research and Technology 8 (2019): 6354–6374, 10.1016/j.jmrt.2019.09.068.

[15] Y. Zhang , Y. Gu , X. Dong , et al., “Deep Oxidative Desulfurization of Refractory Sulfur Compounds With Cesium Salts of Mono‐Substituted Phosphomolybdate as Efficient Catalyst,” Catalysis Letters 147 (2017): 1811–1819, 10.1007/s10562-017-2078-5.

[16] M. Simões , C. Conceição , J. Gamelas , et al., “Keggin‐Type Polyoxotungstates as Catalysts in the Oxidation of Cyclohexane by Dilute Aqueous Hydrogen Peroxide,” Journal of Molecular Catalysis A: Chemical 144 (1999): 461–468.

[17] C. M. Tourné , G. F. Tourné , S. Malik , and T. Weakley , “Triheteropolyanions Containing Copper (II), Manganese (II), or Manganese (III),” Journal of Inorganic and Nuclear Chemistry 32 (1970): 3875–3890.

[18] D. C. Marcano , D. V. Kosynkin , J. M. Berlin , et al., “Improved Synthesis of Graphene Oxide,” ACS Nano 4 (2010): 4806–4814, 10.1021/nn1006368. 20731455

[19] Y. Si and E. T. Samulski , “Exfoliated Graphene Separated by Platinum Nanoparticles,” Chemistry of Materials 20 (2008): 6792–6797, 10.1021/cm801356a.

[20] M. Khalaji‐Verjani and M. Masteri‐Farahani , “Designing a Hybrid Nanomaterial Based on Cr‐Containing Polyoxometalate and Graphene Oxide as an Electrocatalyst for the Hydrogen Evolution Reaction,” Dalton Transactions 53 (2024): 6920–6931, 10.1039/D4DT00320A. 38563196

[21] V. Brusko , A. Khannanov , A. Rakhmatullin , and A. M. Dimiev , “Unraveling the Infrared Spectrum of Graphene Oxide,” Carbon 229 (2024): 119507, 10.1016/j.carbon.2024.119507.

[22] M. Khalaji‐Verjani and M. Masteri‐Farahani , “Selective Photocatalytic Reduction of Nitrobenzenes by H_4_PVMo_11_O_40_ Polyoxometalate Grafted on Graphene Oxide,” Colloids and Surfaces A: Physicochemical and Engineering Aspects 727 (2025): 138463, 10.1016/j.colsurfa.2025.138463.

[23] T. Okuhara , N. Mizuno , and M. Misono , “Catalytic Chemistry of Heteropoly Compounds,” Advances in Catalysis 41 (1996): 113–252, 10.1016/S0360-0564(08)60041-3.

[24] M. Moghadam , V. Mirkhani , S. Tangestaninejad , I. Mohammadpoor‐Baltork , and M. M. Javadi , “Molybdenum Schiff Base‐Polyoxometalate Hybrid Compound: A Heterogeneous Catalyst for Alkene Epoxidation With Tert‐BuOOH,” Polyhedron 29 (2010): 648–654, 10.1016/j.poly.2009.09.016.

[25] S. Aghajani , M. Mohammadikish , and M. Khalaji‐Verjani , “Reusable and Highly Active Copper‐Based Lacunary‐Type Polyoxometalate (LPMo‐Cu) as an Effective Catalyst for Nitroarene Reduction in Aqueous Solution,” Langmuir 39 (2023): 8484–8493, 10.1021/acs.langmuir.3c00766. 37280124

[26] S. Pathan and A. Patel , “Novel Heterogeneous Catalyst, Supported Undecamolybdophosphate: Synthesis, Physico‐Chemical Characterization and Solvent‐Free Oxidation of Styrene,” Dalton Transactions 40 (2011): 348–355, 10.1039/C0DT01187H. 21113531

[27] L. Sun , “Structure and Synthesis of Graphene Oxide,” Chinese Journal of Chemical Engineering 27 (2019): 2251–2260, 10.1016/j.cjche.2019.05.003.

[28] M. Khalaji‐Verjani , M. Masteri‐Farahani , M. Mohammadikish , and M. H. Mashhadizadeh , “Lacunary Polyoxometalate iImmobilized on Graphene Oxide: An Effective Electrocatalyst for Hydrogen Generation in Aqueous Solution,” ChemNanoMat 9 (2023): 202300036, 10.1002/cnma.202300036.

[29] S. Shahsavarifar , M. Masteri‐Farahani , and M. R. Ganjali , “New Water Oxidation Electrocatalyst Based on the Cobalt‐Containing Polyoxometalate‐Reduced Graphene Oxide Hybrid Nanomaterial,” Langmuir 37 (2021): 1925–1931, 10.1021/acs.langmuir.0c03418. 33512170

[30] M. Masteri‐Farahani and M. Modarres , “Clicked Graphene Oxide Supported venturello Catalyst: A New Hybrid Nanomaterial as Catalyst for the Selective Epoxidation of Olefins,” Materials Chemistry and Physics 199 (2017): 522–527, 10.1016/j.matchemphys.2017.07.028.

[31] K. N. Kudin , B. Ozbas , H. C. Schniepp , R. K. Prud'Homme , I. A. Aksay , and R. Car , “Raman Spectra of Graphite Oxide and Functionalized Graphene Sheets,” Nano Letters 8 (2008): 36–41, 10.1021/nl071822y. 18154315

[32] X. H. Li , W. L. Chen , H. Q. Tan , et al., “Reduced State of the Graphene Oxide@ Polyoxometalate Nanocatalyst Achieving High‐Efficiency Nitrogen Fixation Under Light Driving Conditions,” ACS Applied Materials & Interfaces 11 (2019): 37927–37938, 10.1021/acsami.9b12328. 31549811

[33] M. Sharma , J. H. Jang , D. Y. Shin , et al., “Work Function‐Tailored Graphene via Transition Metal Encapsulation as a Highly Active and Durable Catalyst for the Oxygen Reduction Reaction,” Energy & Environmental Science 12 (2019): 2200–2211, 10.1039/C9EE00381A.

[34] W. Chen , H. Li , Y. Jin , et al., “An Intriguing Tetranuclear Rh‐Based Polyoxometalate for the Reduction of Nitroarene and Oxidation of Aniline,” Chemical Communications 58 (2022): 9902–9905, 10.1039/D2CC03076D. 35975716

[35] S. Zhang , Y. Sun , W. Chen , et al., “Facile Synthesis of a Ru‐Based Polyoxometalate for Efficient Reduction of Nitrobenzene,” Dalton Transactions 54 (2025): 9352–9358, 10.1039/D5DT00020C. 40407012

[36] Q. Liu , Y. Zhong , H. Fu , R. Wang , and L. Zhu , “Synergy Effect of Highly Dispersed Palladium and Ni‐SiW Polyoxometalate‐Based Metal‐Organic Framework (POMOF) for Highly Efficient and Selective Nitroaromatics Hydrogenation,” Applied Catalysis A: General 665 (2023): 119373, 10.1016/j.apcata.2023.119373.

[37] S. K. Shi , X. Li , H. L. Guo , et al., “Ionothermal Synthesis of an Antimonomolybdate Cluster,[Sb_8_Mo^VI^ _13_MoV_5_O_66_]^5−^, and Its Catalytic Behavior to the Reduction of Nitrobenzene,” Inorganic Chemistry 59 (2020): 11213–11217. 10.1021/acs.inorgchem.0c00825 32706960

[38] C. Ren , Z. Lu , B. Luo , X. Yi , L. Lin , and L. Xu , “Deeply Reduced Empty Keggin Clusters [Mo^IV^ _x_ M^VI^ _12− x_ O_40− x_ py_x_](x = 3, 6; M = Mo, W; py = pyridine): Synthesis, Structures, and Lewis Field Catalysis,” Inorganic Chemistry Frontiers 8 (2021): 5178–5185, 10.1039/D1QI01080H.

[39] B. Leipzig , “Über Stufenweise Reduktion des Nitrobenzols mit Begrenztem Kathodenpotential,” Zeitschrift für Elektrochemie 4 (1898): 506–514, 10.1002/bbpc.18980042204.

[40] D. Formenti , F. Ferretti , F. K. Scharnagl , and M. Beller , “Reduction of Nitro Compounds Using 3d‐Non‐Noble Metal Catalysts,” Chemical Reviews 119 (2018): 2611–2680, 10.1021/acs.chemrev.8b00547. 30516963

[41] B. Gholami , M. Mohammadikish , and M. Niakan , “Stepwise Growth of Copper‐Based Coordination Polymer on α‐MnO_2_ Nanorods for Sustainable Reduction of Nitroarenes,” ACS Applied Nano Materials 6 (2023): 2864–2872, 10.1021/acsanm.2c05254.

[42] S. Aghajani and M. Mohammadikish , “Sustainable Coordination Polymer‐Based Catalyst and Its Application in the Nitroaromatic Hydrogenation Under Mild Conditions,” Langmuir 38 (2022): 8686–8695, 10.1021/acs.langmuir.2c01208. 35802934

[43] M. Yuan , H. Zhang , C. Yang , F. Wang , and Z. Dong , “Co‐MOF‐Derived Hierarchical Mesoporous Yolk‐Shell‐Structured Nanoreactor for the Catalytic Reduction of Nitroarenes With Hydrazine Hydrate,” Chemcatchem 11 (2019): 3327–3338, 10.1002/cctc.201900714.

