# Supplementary material for: Graphene Oxide‐Supported Chromium‐Containing Polyoxometalate as an Efficient Heterogeneous Catalyst for Nitroarene Reduction Under Mild Conditions
Source: Glob Chall. 2026 Sep 22;10(9):e70159. doi: 10.1002/gch2.70159 (PMC13598426; doi:10.1002/gch2.70159)
Supplement: Supplementary file 1 [file GCH2-10-e70159-s001.docx]

**Supporting Information**

**Graphene Oxide-Supported Chromium-Containing Polyoxometalate as an Efficient Heterogeneous Catalyst for Nitroarene Reduction under Mild Conditions**

Maryam Khalaji-Verjani ^a^, Majid Masteri-Farahani^b^, Alireza Badiei^a,*^, Ghodsi Mohammadi Ziarani^c^, Senem Akkoc^d, e*^, Mehran Feizi-Dehnayebi^f^

^a^ School of Chemistry, College of Science, University of Tehran, Tehran, Iran

^b^ Faculty of Chemistry, Kharazmi University, 15719-14911 Tehran, Iran

^c^ Department of Organic Chemistry, Faculty of Chemistry, Alzahra University, Tehran P.O. Box 1993893973, Iran

^d^Department of Basic Pharmaceutical Sciences, Faculty of Pharmacy, Suleyman Demirel University, Isparta, Türkiye

^e^Faculty of Engineering and Natural Sciences, Bahcesehir University, Istanbul, Türkiye

^f^ Department of Chemical Engineering, Engineering Faculty, Suleyman Demirel University, Isparta, Turkey

*Corresponding authors E-mail:

* [abadiei@ut.ac.ir](mailto:abadiei@ut.ac.ir)

* [senemakkoc@sdu.edu.tr](mailto:senemakkoc@sdu.edu.tr)

**S1. Experimental**

All chemicals employed in this study were purchased from Sigma-Aldrich, Arcos, Merck, or Fluka and used as received without additional purification.

**Synthesis of graphene oxide (GO)**

The first, 1 g of graphite powder was mixed with 120 mL of concentrated H₂SO₄, and the stirred for 10 minutes. Then, 6 g of KMnO₄ was slowly added to the mixture over 30 minutes. The reaction was heated to 50-60 °C and maintained under vigorous stirring for 24 hours. After the reaction period, 130 mL of deionized (DI) water and 3 mL of a 30% hydrogen peroxide were added to neutralize excess KMnO_4_ and oxidize any remaining manganese species. Finally, 250 mL of a 5% HCl was added to dissolve insoluble manganese oxides and residual salts [1].

**S2. Characterization**

The structural characteristics of the synthesized catalyst and related compounds were investigated using several analytic techniques. Fourier-transform infrared (FTIR) spectra of the solid samples were obtained using a Perkin-Elmer RXI FT-IR spectrometer with KBr pellets. Powder X-ray diffraction (PXRD) patterns were recorded on an X'Pert Pro instrument employing Cu Kα radiation. The morphological features and elemental composition were analyzed by field-emission scanning electron microscopy (FE-SEM) coupled with energy-dispersive X-ray spectroscopy (EDX) on a TESCAN MIRA III system, providing detailed microstructural mapping. Specific surface area measurments were performed via the Brunauer-Emmett-Teller (BET) N₂ adsorption method using a BElSORP Mini/Microtrac BEL Corp analyzer (Japan). Reaction mechanisms and catalytic performance were assessed by gas chromatography-mass spectrometry (GC-MS; Agilent 7890A-MSD), while reaction progress was monitored by thin-layer chromatography (TLC) using an ethyl acetate/n-hexane (1:6 v/v) as the mobile phase.

**S3. GC-MS analysis**

**Fig. S1.** The GC-MS of the products from 1-Methoxy-4-nitrobenzene reduction reaction over 2 h.

**S4. Abbreviations**

| POM | Polyoxometalates |
| --- | --- |
| Na_4_[PCr(H_2_O)Mo_11_O_39_] | PMo_11_Co |
| Transition Metal Substituted Polyoxometalates | TMSPOMs |
| APTS | Amino propyl trimethoxy silane |
| NB | Nitrobenzene |
| GO | Graphen oxide |
| GC-MS | Gas Chromatography- mass spectrometry |
| XRD | X-ray Diffraction |
| FESEM | Field-Emission Scanning Electron Microscope |
| FT-IR | Fourier transforms infrared spectroscopy |
| BET | Brunauer-Emmett-Teller |
| TLC | Thin-layer chromatography |

**References**

1.	Marcano, D.C., et al., Improved synthesis of graphene oxide. ACS Nano, 2010. **4**(8): p. 4806-4814.
